# Supplementary material for: Inactivation of SARS-CoV-2 at acidic pH is driven by partial unfolding of spike
Source: Commun Biol. 2025 Jul 21;8:1082. doi: 10.1038/s42003-025-08514-w (PMC12280015; doi:10.1038/s42003-025-08514-w)
Supplement: Supplementary file 2 — Supplementary Information [file 42003_2025_8514_MOESM2_ESM.pdf]

Supplementary Information

Inactivation of SARS-CoV-2 at acidic pH is driven by partial unfolding of spike

Irina Glas[1], Liv Zimmermann[2,3], Beiping Luo[4], Marie O. Pohl[1], Antoni G. Wrobel[5], Aline Schaub[6], Liviana K. Klein[4], Shannon C. David[6], Elisabeth Gaggioli[1], Nir Bluvshtein[4], Michael Huber[1], Athanasios Nenes[7,8], Ulrich K. Krieger[4], Thomas Peter[4], Tamar Kohn[6], Petr Chlanda[2,3] and Silke Stertz[1]*

[1] Institute of Medical Virology, University of Zurich, Zurich, Switzerland

[2] Schaller Research Group, Department for Infectious Diseases, Virology, Heidelberg University, Heidelberg, Germany

[3] BioQuant - Research Center for Quantitative Analysis of Molecular and Cellular Systems, Heidelberg University, Heidelberg, Germany

[4] Institute for Atmospheric and Climate Science, ETH Zurich, Zurich, Switzerland

[5] Department of Biochemistry, University of Oxford, Oxford, United Kingdom

[6] Environmental Chemistry Laboratory, School of Architecture, Civil and Environmental Engineering, Swiss Federal Institute of Technology in Lausanne, Lausanne, Switzerland

[7] Laboratory of Atmospheric Processes and their Impacts, School of Architecture, Civil and Environmental Engineering, Swiss Federal Institute of Technology in Lausanne, Lausanne, Switzerland

[8] Institute of Chemical Engineering Sciences, Foundation for Research and Technology Hellas, Patras, Greece

* Corresponding author: Silke Stertz, Ph.D. (E-mail: stertz.silke@virology.uzh.ch)

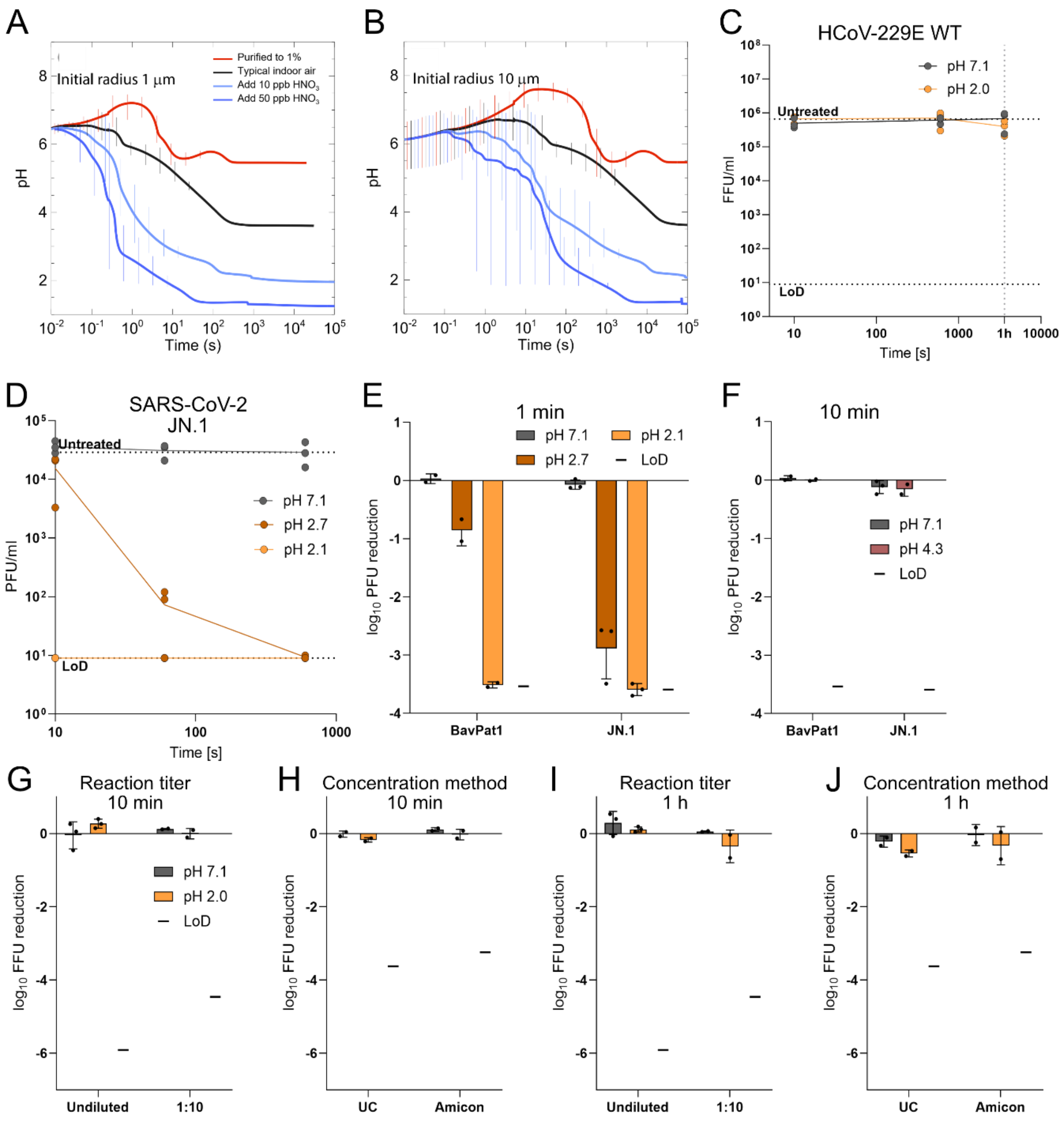

Figure S1: A and B) ResAM simulation of the average IRP pH over time in particles with a 1 µm (A) or 10 µm (B) radius upon exhalation. Differing air compositions including typical indoor air, purified air removing 99% of HNO_3 (purified to 1%) and acidified air with higher HNO_3 content (of 10 ppbv HNO_3 and of 50 ppbv HNO_3) were simulated. Error bars indicate the pH range within a single IRP, i.e. the minimum and maximum pH calculated by ResAM for the various shells within the particle. Indoor air parameters were set to 20 °C and 50 % RH. **C)** Inactivation curves in FFU/ml of HCoV-229E wild type (HCoV-229E WT), over time at pH 7.1 and 2.0. Data was derived from three independent experiments. Dotted lines indicate the titer measured in the untreated control and the LoD. **D)** Inactivation curves in PFU/ml of SARS-CoV-2 JN.1 Omicron isolate, over time at pH 7.1, 2.7 and 2.1. Data was derived from three independent experiments. Dotted lines indicate the titer measured in the untreated control and the LoD. **E)** Calculated log_{10} loss of PFU/ml normalized to the pH 7.1 10 s sample of the data shown in C) after 1 min of pH treatment (JN.1).

We additionally show inactivation data of SARS-CoV-2 BavPat1 samples diluted to the same reaction titer as SRAS-CoV-2 JN.1 after 1 min of exposure to acidic pH. Data was derived from n = 2 and n =3 independent experiments for SARS-CoV-2 BavPat1 and JN.1 respectively. **F)** Calculated log_{10} reduction in PFU/ml of SARS-CoV-2 BavPat1 and JN.1 at pH 7.1 and 4.3 after 10 min of exposure normalized to the control (pH 7.1 for 10 s). Data was derived from n = 2 independent experiments. **G-J)** Calculated log_{10} reduction in PFU/ml of HCoV-229E-Ren exposed to pH 7.1 or 2.0 for 10 min (G and H) and 1 h (I and J) normalized to the control (pH 7.1 10 s). We compared the loss of PFU/ml of different titers in the pH inactivation reaction (G and I). Shown is the log_{10} loss calculated from the data in figure 1D (undiluted) as well as from samples with a 10-fold lower virus concentration in the reaction (comparable to the reaction titer of SARS-CoV-2 BavPat1 in figure 1B). Further, we compare the impact of virus concentration methods (H and J). HCoV-229E-Ren was either concentrated by ultracentrifugation (UC) or with Amicon tubes (Amicon) and diluted to the same titer before exposure to acidic pH. Data was derived from two to three independent replicates (n = 2 per group, except “undiluted” groups where n =3). Grey dashes report the LoD for each set of inactivation experiments. Data in E-J are means with the error bars representing SD.

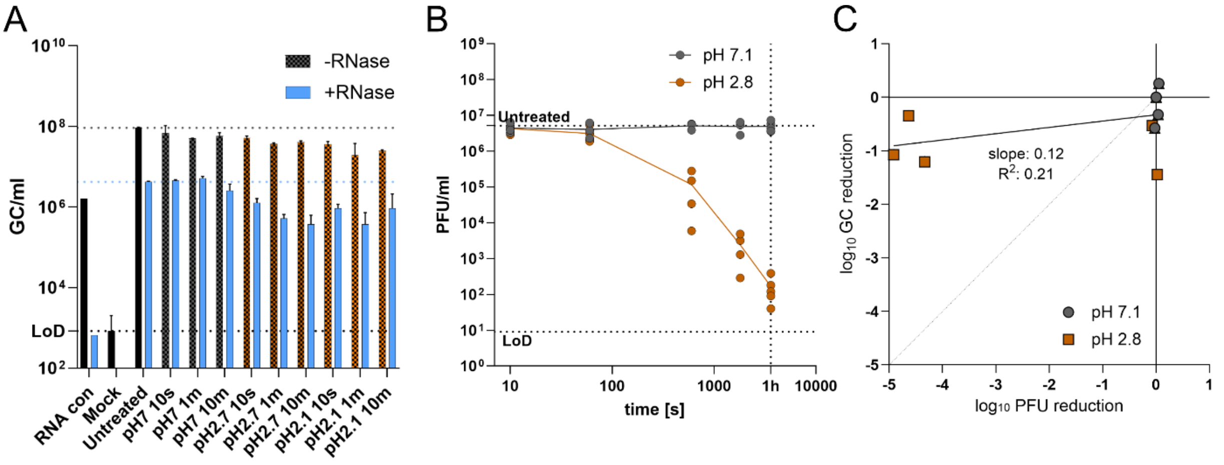

Figure S2: **A)** Raw data in genomic copies (GC)/ml from one representative SARS-CoV-2 BavPat1 replicate of an RNase digestion assay. GC were measured by qPCR. Samples not exposed to RNase are shown in black (-RNase), while RNase digested samples are shown in blue (+RNase). Error bars represent the SD from the mean of n = 3 qPCR replicates. The dashed lines show the limit of detection (LoD), the GC of the untreated control with and without RNase (blue and black accordingly). **B)** Inactivation curve in PFU/ml of SARS-CoV-2 BavPat1 at acidic pH from at least three independent experiments per time point. To obtain higher titers for certain experiments we increased the amount of virus stock added into the pH reaction, which led to a slower inactivation dynamic. Dashed lines show the LoD, the measured titer of the untreated control (untreated) and the 1 h time point. **C)** Correlation of log_{10} loss in GC/ml and PFU/ml of SARS-CoV-2 BavPat1 samples with increased titer and protein concentration treated with pH 7.1 or pH 2.8. Linear regression was performed using GraphPad Prism (n = 11). The coefficient of determination (R^2) is 0.21. The slope is 0.12 (95% CI: [0.05, 0.29]), with a p-value of 0.15. Dotted line represents a perfect correlation.

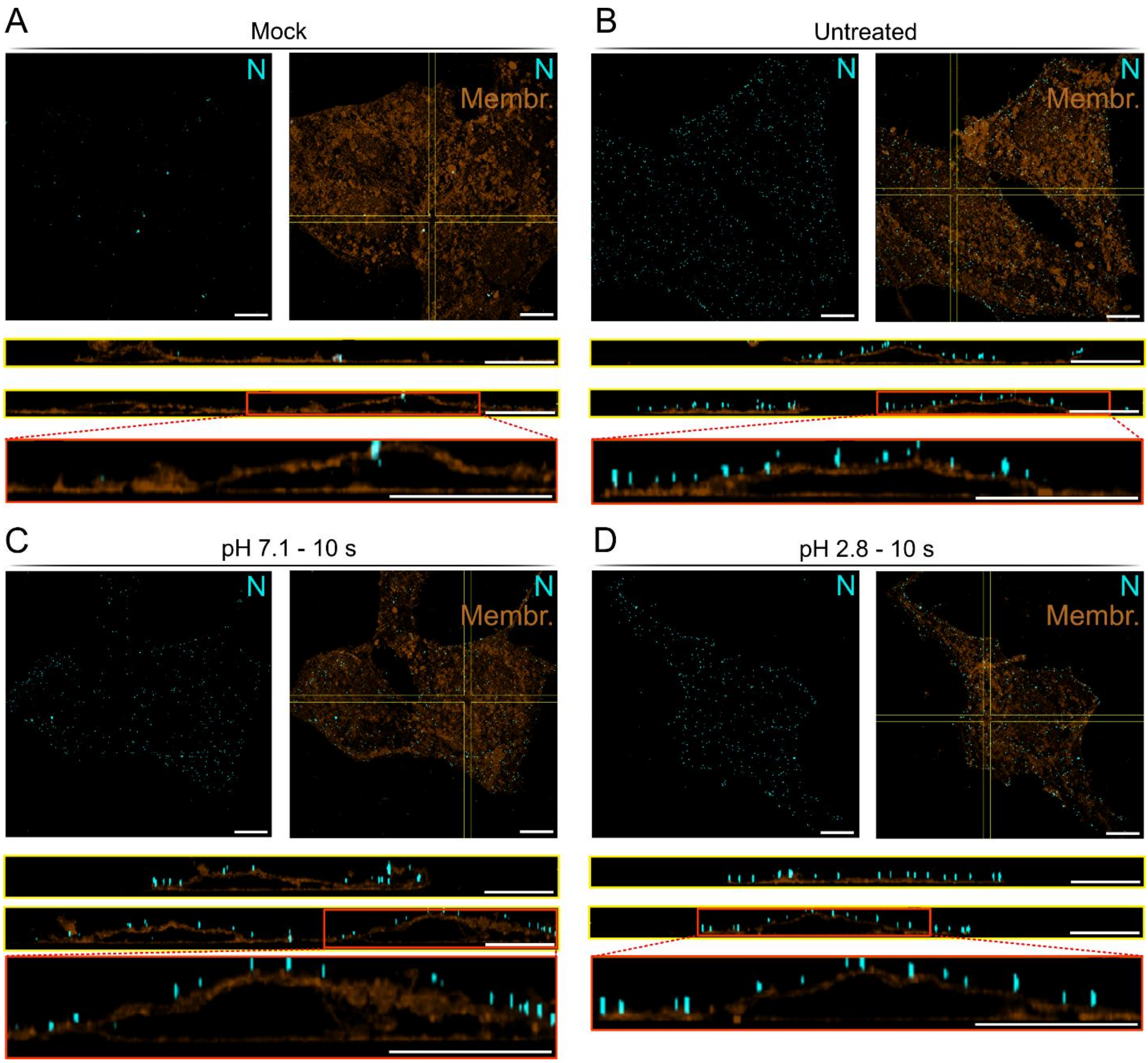

Figure S3: Binding assays with the same experimental procedure as described in Figure 3. **A and B)** Z-stacks of cells from the uninfected mock (A) and untreated SARS-CoV-2 BavPat1 samples (B). Representative images were chosen from three independent experiments with each four to eight z-stacks per condition. **C and D)** Z-stacks of cells incubated with SARS-CoV-2 BavPat1 samples exposed to pH 7.1 or pH 2.8 for 10 s (C and D respectively). Representative images were chosen from six to eight z-stacks generated in a single experiment. Scale bars correspond to 10 µm.

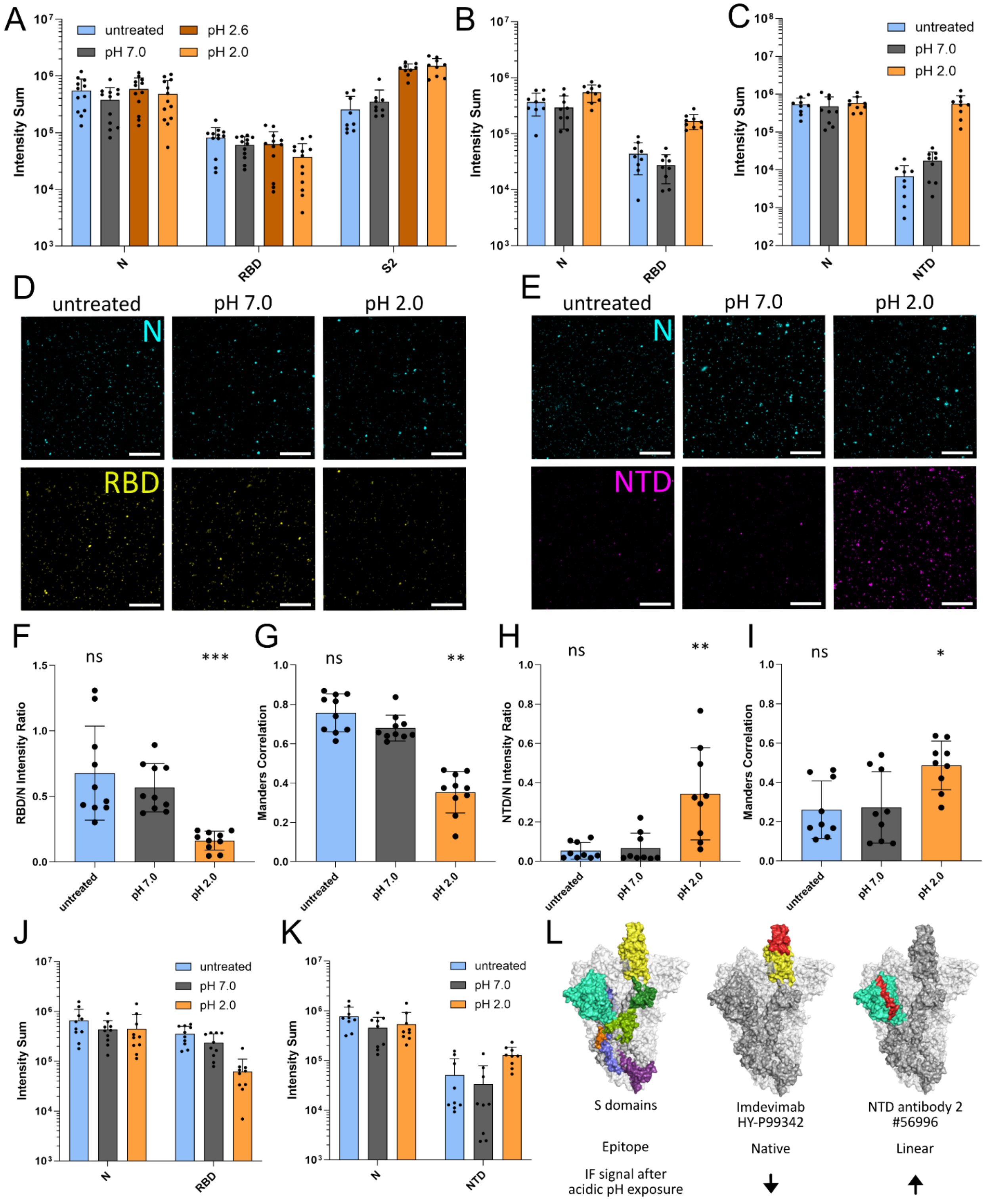

Figure S4: **A-C)** Intensity sums of the analyzed images in Figure 5D-G (A), Figure 5H and I (B) and Figure 5J and K (C). Only pixels positive for N signal were considered in the analysis. Three to four images were analyzed per condition derived from three to four independent experiments (n = 9 per group, except the N and RBD intensity sums in A where n = 12 for undiluted, pH 7.0 and pH 2.6 and n = 13 for pH 2.0). Error

bars represent SD from the mean. **D and E)** As in figure 5A-C, virions were treated with acidic pH and stained with S antibodies and an N antibody as a control (cyan). We confirmed our results with one additional RBD antibody (Imdevimab, HY-P99342) (D) and one additional NTD antibody (#56996) (E). Shown are representative images of three independent experiments. Scale bar corresponds to 10 µm. **F-I)** Quantification of three to four images from three independent experiments as described for figure 5D-K (n = 10 per group in F and G and n= 9 per group in H and I). The signal intensity ratio of the Imdevimab (F) or NTD #56996 antibody (H) to the N antibody signal was calculated for each image. The Manders correlation for Imdevimab (G), and the second NTD antibody (I) was determined using Coloc 2 (ImageJ). Error bars represent SD from the mean. All statistical tests were done with a nonparametric one-way ANOVA (Kruskal-Wallis test, two-sided) in GraphPad Prism comparing all conditions to the pH 7.0 control. Data was considered non-significant (ns) if p values were 0.05 or above and significant for p values below 0.05 ($p < 0.05$ (*), $p < 0.005$ (**), $p < 0.0005$ (***)). **J and K)** Intensity sums of N positive pixels from images analyzed in F-I (same n values as in F-I). Shown are intensity sums of samples stained with Imdevimab (J) and images stained with the NTD antibody #56996 (K). Error bars represent SD from the mean. **L)** As in figure 5N, antibody epitopes were visualized on an S trimer (PDB ID: 6ZGG). For each antibody, the epitope preference is shown (linear or native) and how the antibody binding was affected after pH treatment as determined by the IF signal. S domains were colored as follows: NTD (turquois), RBD (yellow), CTD1 (dark green), CTD2 (light green), fusion peptide (orange), central helix (dark blue), heptad repeat 1 (HR1, light blue) and HR2 (purple). For each antibody, the epitope was colored red and overlapping domains were colored as assigned. Imdevimab binds the RBM amino acids (AAs) 438-506 within the RBD (yellow). NTD antibody #56996 binds a synthetic peptide in the NTD (turquois). Since the immunogen is a peptide surrounding Pro25, AAs 14 to 50 were colored red. Of note, AAs before AA 14 were not resolved in the structure 6ZGG. In A, B and E-J data are means with the error bars representing SD.

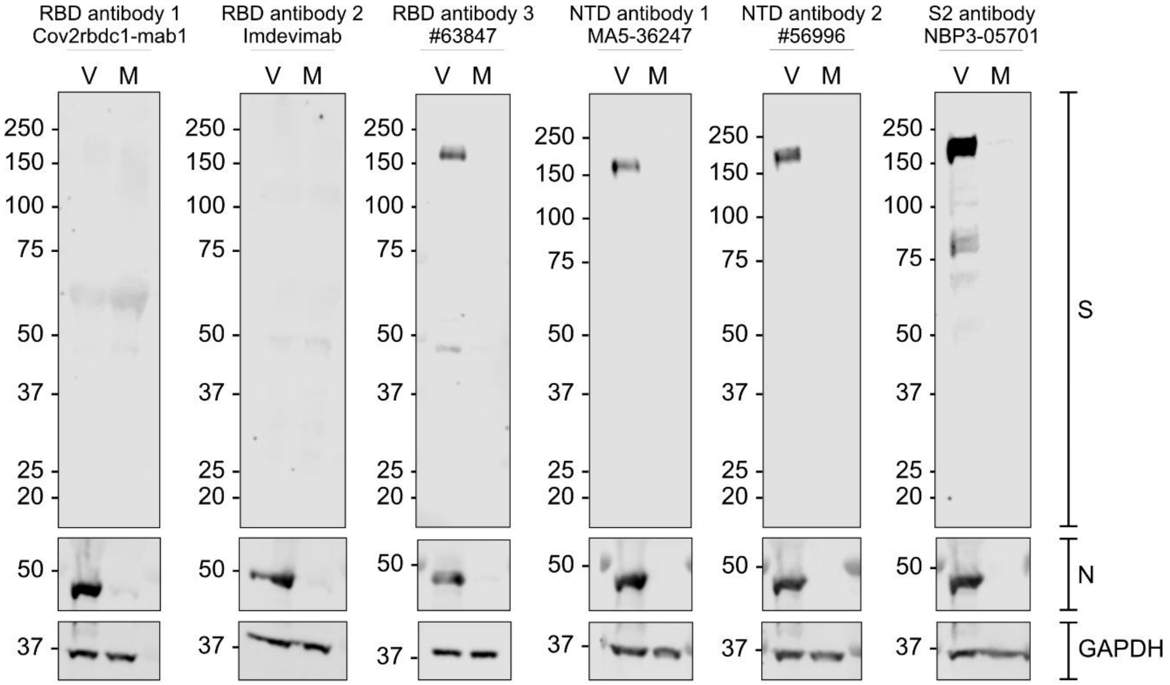

Figure S5: Western blots with all SARS-CoV-2 BavPat1 S antibodies used in figure 5 and S4. VAT cells were infected with BavPat1 (V) or a mock control (M) for 16 h. Lysates of infected cells were then used for testing different S antibodies for their performance in WB. All S antibodies were applied in a 1:1,000 dilution. Anti-N (MA5-29981) and anti-GAPDH (sc-25778) antibodies were used to control for viral infection and cell lysate loading on gels.

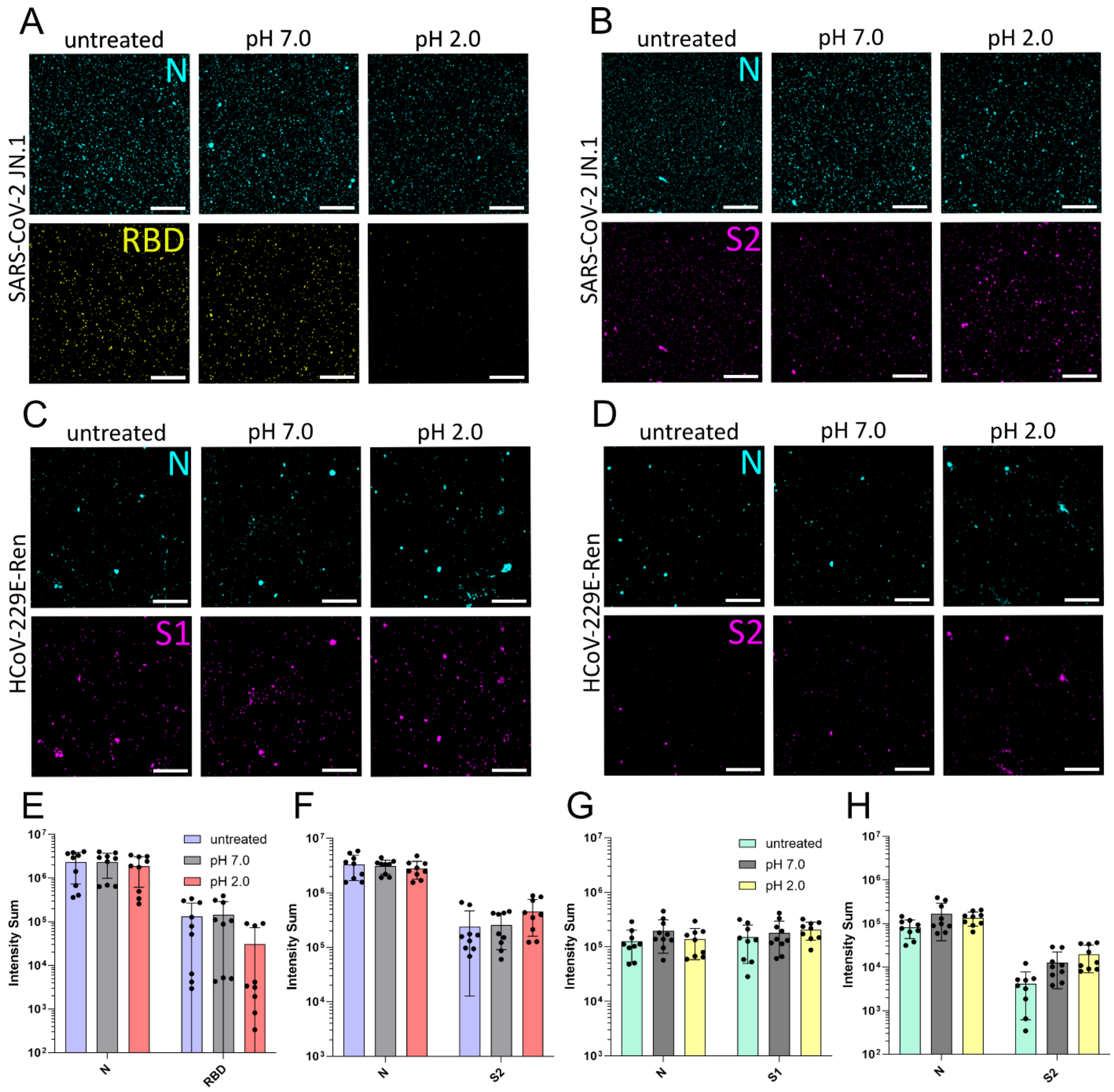

Figure S6: A-D) SARS-CoV-2 JN.1 or HCoV-229E-Ren virions were treated with acidic pH and stained with S antibodies and an N antibody as a control (cyan). For SARS-CoV-2 JN.1 we chose a neutralizing RBD antibody (MA5-47208) (A) and the S2 antibody (NBP3-05701) (B) and for HCoV-229E-Ren we selected an S1 antibody (40601-T62) (C) and S2 antibody (PA5120721) (D). Shown are representative images of three independent experiments. Scale bar corresponds to 10 µm. **E-H)** Intensity sums of the analyzed images in figure 6A and B (E), figure 6C and D (F), figure 6E and F (G) and figure 6G and H (H). Only pixels positive for N signal were considered in the analysis. Three images were analyzed per condition derived from three independent experiments (n = 9 per group). Error bars represent SD from the mean.

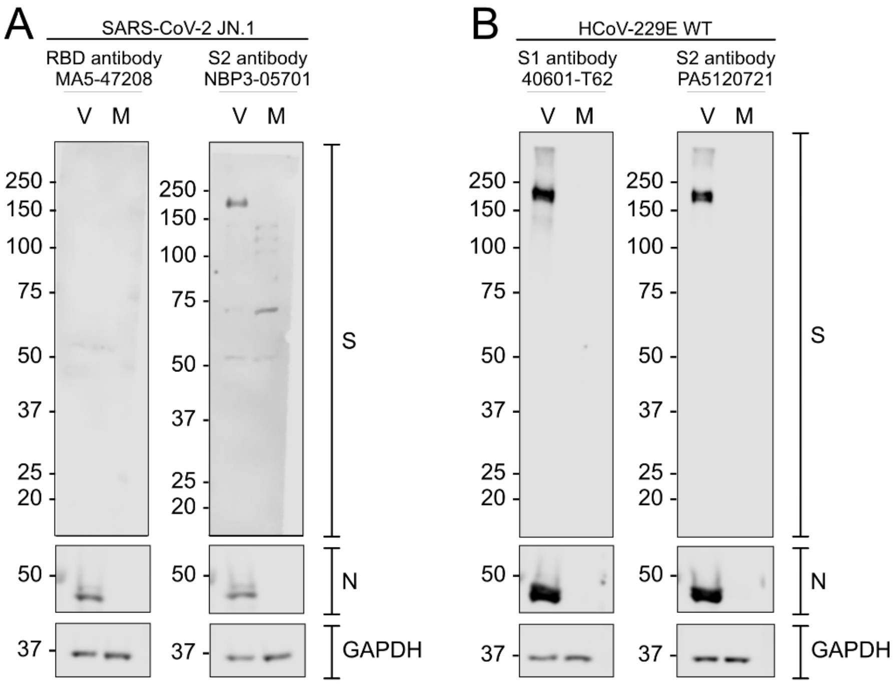

Figure S7: Western blots with all SARS-CoV-2 JN.1 and HCoV-229E S antibodies used in figure 6. **A)** VAT cells were infected with SARS-CoV-2 JN.1 (V) or a mock control (M) for 20 h. Lysates of infected cells were then used for testing different S antibodies for their performance in WB. All S antibodies were applied in a 1:1,000 dilution. Anti-N (MA5-29981) and anti-GAPDH (sc-25778) antibodies were used to control for viral infection and cell lysate loading on gels. **B)** As in A, but with Huh-7 cells infected with HCoV-229E WT for 28 h. An HCoV-229E anti-N antibody (40640-MM11) was used to control for infection with HCoV-229E WT.

WB from figure 5L

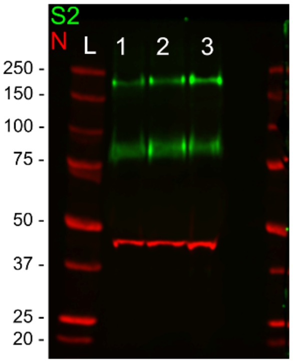

WBs from figure S5

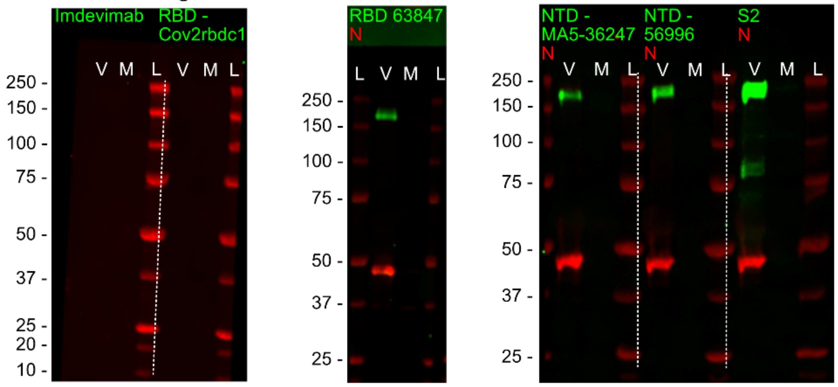

Membranes were cut above the 50 kDa mark and stained for GAPDH (37 kDa)

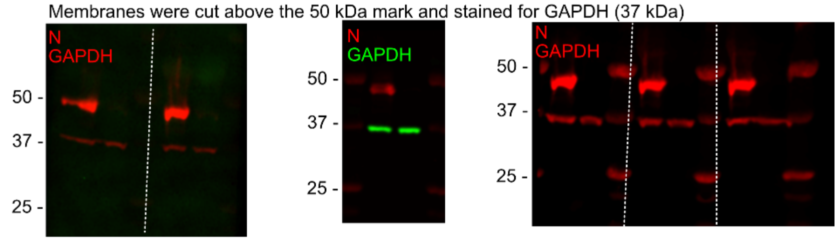

WBs from figure S7

VAT cells and JN.1

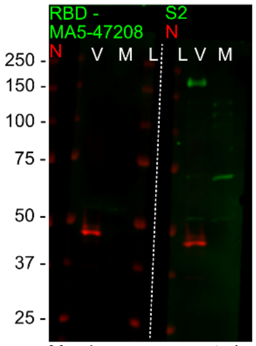

Huh-7 cells and 229E

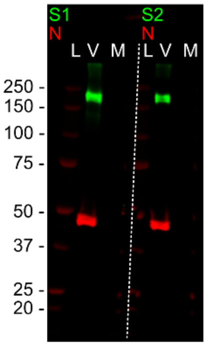

Membranes were cut above the 50 kDa mark and stained for GAPDH (37 kDa)

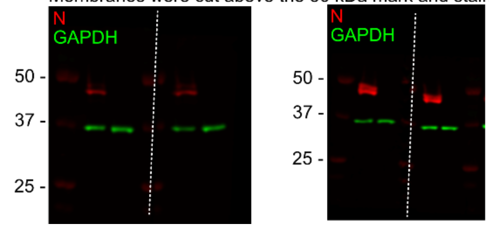

Figure S8: Full WBs from figure 5, S5 and S7. Dotted line indicates were membranes were cut to allow simultaneous staining with different antibodies. Note that some membranes were cut to stain with an additional third GAPDH antibody as indicated in the figure. Lanes with infected cells were marked with a "V", mock infected cells with "M" and ladders with "L".
