# Supplementary material for: Inactivation of SARS-CoV-2 at acidic pH is driven by partial unfolding of spike
Source: Commun Biol. 2025 Jul 21;8:1082. doi: 10.1038/s42003-025-08514-w (PMC12280015; doi:10.1038/s42003-025-08514-w)
Supplement: Supplementary file 3 — Description of Additional Supplementary Files [file 42003_2025_8514_MOESM3_ESM.docx]

Description of Additional Supplementary Files

**File name:** Supplementary Data 1

**Description:** Results for all peptides identified in LiP LC-MS.

**File name:** Supplementary Data 2

**Description:** Source data and p values underlying the graphs in the paper.
