# Supplementary material for: Inactivation of SARS-CoV-2 at acidic pH is driven by partial unfolding of spike
Source: Commun Biol. 2025 Jul 21;8:1082. doi: 10.1038/s42003-025-08514-w (PMC12280015; doi:10.1038/s42003-025-08514-w)
Supplement: Supplementary file 6 — Reporting Summary [file 42003_2025_8514_MOESM6_ESM.pdf]

nature portfolio

Corresponding author(s): Stertz, Silke

Last updated by author(s): Jul 2, 2025

Reporting Summary

Nature Portfolio wishes to improve the reproducibility of the work that we publish. This form provides structure for consistency and transparency in reporting. For further information on Nature Portfolio policies, see our Editorial Policies and the Editorial Policy Checklist.

Statistics

For all statistical analyses, confirm that the following items are present in the figure legend, table legend, main text, or Methods section.

n/a	Confirmed	
☐	☒	The exact sample size (*n*) for each experimental group/condition, given as a discrete number and unit of measurement
☐	☒	A statement on whether measurements were taken from distinct samples or whether the same sample was measured repeatedly
☐	☒	The statistical test(s) used AND whether they are one- or two-sided<br>*Only common tests should be described solely by name; describe more complex techniques in the Methods section.*
☒	☐	A description of all covariates tested
☒	☐	A description of any assumptions or corrections, such as tests of normality and adjustment for multiple comparisons
☐	☒	A full description of the statistical parameters including central tendency (e.g. means) or other basic estimates (e.g. regression coefficient) AND variation (e.g. standard deviation) or associated estimates of uncertainty (e.g. confidence intervals)
☐	☒	For null hypothesis testing, the test statistic (e.g. *F*, *t*, *r*) with confidence intervals, effect sizes, degrees of freedom and *P* value noted<br>*Give P values as exact values whenever suitable.*
☒	☐	For Bayesian analysis, information on the choice of priors and Markov chain Monte Carlo settings
☒	☐	For hierarchical and complex designs, identification of the appropriate level for tests and full reporting of outcomes
☒	☐	Estimates of effect sizes (e.g. Cohen's *d*, Pearson's *r*), indicating how they were calculated

Our web collection on statistics for biologists contains articles on many of the points above.

Software and code

Policy information about availability of computer code

Data collection	PACEtomo v1.6.1, SerialEM v4.1.0 beta, MotionCor2 1.3.1, IMOD v5.0.1, 7300 System SDS Software v1.4, Leica LAS X software v3.0.2.16120, Zeiss ZEN black edition 3.0
Data analysis	7300 System SDS Software v1.4, GraphPad Prism 10.2.3, ImageJ 2.14.0 (Java 1.8.0_172), Zeiss ZEN black edition 3.0, Bitplane Imaris v10.1.1, Li-Cor Image Studio Lite v5.2, Spectronaut (19.9.250422.62635)

For manuscripts utilizing custom algorithms or software that are central to the research but not yet described in published literature, software must be made available to editors and reviewers. We strongly encourage code deposition in a community repository (e.g. GitHub). See the Nature Portfolio guidelines for submitting code & software for further information.

Data

Policy information about availability of data

All manuscripts must include a data availability statement. This statement should provide the following information, where applicable:

- Accession codes, unique identifiers, or web links for publicly available datasets
- A description of any restrictions on data availability
- For clinical datasets or third party data, please ensure that the statement adheres to our policy

All data, with the exception of the LiP LC-MS data, supporting the findings of this study are available within the paper and its supplementary information files. LiP LC-

MS data have been deposited to the ProteomeXchange Consortium via the PRIDE partner repository with the dataset identifier PXD064612. The sequence of the SARS-CoV-2 JN.1 isolate is available on GenBank (PV414078.1)

Research involving human participants, their data, or biological material

Policy information about studies with human participants or human data. See also policy information about sex, gender (identity/presentation), and sexual orientation and race, ethnicity and racism.

Reporting on sex and gender	NA
Reporting on race, ethnicity, or other socially relevant groupings	NA
Population characteristics	NA
Recruitment	NA
Ethics oversight	NA

Note that full information on the approval of the study protocol must also be provided in the manuscript.

Field-specific reporting

Please select the one below that is the best fit for your research. If you are not sure, read the appropriate sections before making your selection.

☒ Life sciences ☐ Behavioural & social sciences ☐ Ecological, evolutionary & environmental sciences

For a reference copy of the document with all sections, see nature.com/documents/nr-reporting-summary-flat.pdf

Life sciences study design

All studies must disclose on these points even when the disclosure is negative.

Sample size	No statistical methods were used to pre-determine sample sizes but our sample sizes are similar to those reported in previous publications using similar methods and tools (doi: 10.1128/msphere.00226-23; doi: 10.1128/jvi.00409-24).
Data exclusions	No data were excluded from the analysis.
Replication	For all major experiments at least three independent experiments were done and in all cases results could be reproduced. The number of repeats for each experiment is reported in the figure legends. Limited protealysis LC-MS was performed as one experiment with four technical replicates.
Randomization	Allocation of samples to groups was random.
Blinding	Investigators were not blinded to group allocation in this study as this was a discovery-based study.

Reporting for specific materials, systems and methods

We require information from authors about some types of materials, experimental systems and methods used in many studies. Here, indicate whether each material, system or method listed is relevant to your study. If you are not sure if a list item applies to your research, read the appropriate section before selecting a response.

Materials & experimental systems

n/a	Involved in the study	
☐	☒	Antibodies
☐	☒	Eukaryotic cell lines
☒	☐	Palaeontology and archaeology
☒	☐	Animals and other organisms
☒	☐	Clinical data
☒	☐	Dual use research of concern
☒	☐	Plants

Methods

n/a	Involved in the study	
☒	☐	ChIP-seq
☒	☐	Flow cytometry
☒	☐	MRI-based neuroimaging

Antibodies

Antibodies used	- SARS-CoV-2 Spike Protein (S1-NTD) Antibody, #56996, Cell Signaling, RRID:AB_3492098<br>- SARS/SARS-CoV-2 Nucleocapsid Monoclonal Antibody, MA5-29981, Thermo Fisher Scientific, RRID:AB_2785780<br>- IRDye anti-rabbit 800CW, 926-32211, Li-Cor, RRID:AB_621843<br>- IRDye anti-mouse 680RD, 926-68070, Li-Cor, RRID:AB_10956588<br>- Human coronavirus (HCoV-229E) Nucleocapsid Antibody, Rabbit PAb, Antigen Affinity Purified, 40640-T62 Sino Biological<br>- Anti-Rabbit IgG (whole molecule)–Peroxidase antibody produced in goat, A0545, Sigma-Aldrich, RRID:AB_257896<br>- Imdevimab (Synonyms: REGN10987); HY-P99342, Medchem Express<br>- SARS-CoV-2 Spike RBD monoclonal human IgG1 antibody (Clone H4), cov2rbdc1-mab1, Invivogen<br>- SARS-CoV-2 Spike Protein (RBD) (E7B3E) Rabbit mAb, #63847 Cell Signaling Technology, RRID:AB_3674090<br>- SARS-CoV-2 Spike Protein RBD Omicron Recombinant Rabbit Monoclonal Antibody (HL1867), MA5-47208 Thermo Fisher Scientific, RRID:AB_2938280<br>- SARS-CoV-2 Spike Protein S1 Recombinant Rabbit Monoclonal Antibody (HL6), MA5-36247, Thermo Fisher Scientific, RRID:AB_2890589<br>- SARS-CoV-2 Spike S2 Antibody - BSA Free, NBP3-05701, Novus Bio, RRID:AB_3534207<br>- Human coronavirus (HCoV-229E) Nucleocapsid Antibody, Mouse Mab, 40640-MM11, Sino Biological, RRID:AB_3676911<br>- Human coronavirus (HCoV-229E) Spike S1 Antibody, Rabbit PAb, Antigen Affinity Purified, 40601-T62 Sino Biological<br>- HCoV-229E Spike S2 Polyclonal Antibody, PIPA5120721, Invitrogen, RRID:AB_2914293<br>- GAPDH Antibody (0411), sc-47724, Santa Cruz, RRID:AB_627678<br>- Donkey anti-Mouse IgG (H+L) Highly Cross-Adsorbed Secondary Antibody, Alexa Fluor™ 488, A-21202; Thermo Fisher Scientific, RRID:AB_141607<br>- Goat anti-Human IgG (H+L) Cross-Adsorbed Secondary Antibody, Alexa Fluor 594, A-11014, Thermo Fisher Scientific), RRID:AB_2534081<br>- Donkey anti-Rabbit IgG (H+L) Highly Cross-Adsorbed Secondary Antibody, Alexa Fluor™ 647, A-31573, Thermo Fisher scientific, RRID:AB_2536183
Validation	- The N antibodies (MA5-29981 and 40640-T62) were validated in immunofluorescene by testing on uninfected versus infected samples<br>- S antibodies were validated in western blot by testing them on lysates from uninfected versus infected cells<br>- S antibodies were tested on untreated virus particles in immunofluorescence<br>- Secondary antibodies were tested by including controls without primary antibodies<br>- This study only involves commercial antibodies that were tested by the manufacturers and described on the according website

Eukaryotic cell lines

Policy information about cell lines and Sex and Gender in Research

Cell line source(s)	Huh-7 cells were kindly provided by Georg Kochs, Universitätsklinikum Freiburg, Germany and Vero-E6 cells were kindly provided by Volker Thiel, University of Bern, Switzerland. Vero-E6 cells stably expressing ACE2 and TMPRSS2 (VAT) were kindly provided by Sam Wilson, University of Glasgow, United Kingdom.
Authentication	No cell line authentication was performed.
Mycoplasma contamination	Cell lines used in the Stertz lab are routinely tested for mycoplasma contamination by sending representative samples for mycoplasma testing at GATC, Germany or Microsynth, Switzerland. None of the used cell lines tested positive for mycoplasma.
Commonly misidentified lines (See ICLAC register)	No commonly misidentified cell lines were used.

Plants

Seed stocks	NA
Novel plant genotypes	NA
Authentication	NA
